# Epidemiology of biopsy-proven Henoch–Schönlein purpura nephritis in children: A nationwide survey in Japan

**DOI:** 10.1371/journal.pone.0270796

**Published:** 2022-07-08

**Authors:** Chikako Terano, Riku Hamada, Ichiro Tatsuno, Yuko Hamasaki, Yoshinori Araki, Yoshimitsu Gotoh, Koichi Nakanishi, Hitoshi Nakazato, Takeshi Matsuyama, Kazumoto Iijima, Norishige Yoshikawa, Tetsuji Kaneko, Shuichi Ito, Masataka Honda, Kenji Ishikura, on behalf of the Japanese Study Group of Renal Disease in Children

**Affiliations:** 1 Department of Nephrology, Tokyo Metropolitan Children’s Medical Center, Fuchu, Tokyo, Japan; 2 Department of Diabetes, Endocrinology and Metabolism, Toho University Graduate School of Medicine, Oota-ku, Tokyo, Japan; 3 Center for Diabetes, Endocrinology and Metabolism, Toho University Sakura Medical Center, Sakura, Chiba, Japan; 4 Chiba Prefecture University of Health Science, Mihama-ku, Chiba, Japan; 5 Department of Nephrology, Toho University Faculty of Medicine, Oota-ku, Tokyo, Japan; 6 Department of Pediatric Nephrology, National Hospital Organization Hokkaido Medical Center, Sapporo, Hokkado, Japan; 7 Department of Pediatric Nephrology, Japanese Red Cross Aichi Medical Center Nagoya Daini Hospital, Nagoya, Aichi, Japan; 8 Department of Child Health and Welfare (Pediatrics), Graduate School of Medicine, University of the Ryukyus, Nishihara-cho, Okinawa, Japan; 9 Department of Pediatrics, Faculty of Life Sciences, Kumamoto University, Chuo-ku, Japan; 10 Department of Pediatrics, Fussa Hospital, Fussa, Tokyo, Japan; 11 Department of Nephrology, Hyogo Prefectural Kobe Children’s Hospital, Kobe, Hyogo, Japan; 12 Clinical Research Center, Takatsuki General Hospital, Takatsuki, Oosaka, Japan; 13 Teikyo Academic Research Center, Teikyo University, Itabashi-ku, Tokyo, Japan; 14 Department of Pediatrics, Yokohama City University Hospital, Yokohama, Kanagawa, Japan; 15 Department of Pediatrics, Kitasato University School of Medicine, Sagamihara, Kanagawa, Japan; University of KwaZulu-Natal, SOUTH AFRICA

## Abstract

**Background:**

Little is known about the epidemiology of Henoch–Schönlein purpura nephritis (HSPN).

**Methods:**

We conducted a nationwide epidemiological survey of Japanese children aged 1 to 15 years with HSPN. Children who were newly diagnosed with HSPN by biopsy between January 2013 and December 2015 were eligible for the survey to clarify the incidence of HSPN. We also conducted an institutional survey on kidney biopsy criteria and treatment protocols.

**Results:**

A total of 353 of 412 institutions (85.7%) responded to the questionnaire. Of the 353 institutions, 174 reported to perform kidney biopsies at their institutions, and 563 children were diagnosed with HSPN. Considering the collection rate, the estimated incidence of biopsy-proven HSPN was 1.32 cases/100,000 children per year. The median age at biopsy was 7.0 years, and the male-to-female ratio was 1.2:1. The kidney biopsy criteria and treatment protocols for HSPN were as follows. Patients with acute kidney injury underwent biopsy at least one month after onset. For patients without kidney dysfunction, the timing for biopsy was determined by the amount of proteinuria. Regarding the treatment of HSPN, there were certain commonalities among the treatment protocols, they eventually differed depending on the institutions involved.

**Conclusions:**

The incidence of biopsy-proven HSPN was 1.32 cases/100,000 children per year in Japan. The male-to-female ratio and date of diagnosis of HSPN were similar to those in previous studies. The kidney biopsy criteria and treatment protocols for HSPN varied among institutions. Further studies are warranted to establish an optimal treatment policy based on the prognosis.

## Introduction

IgA vasculitis is an acute small-vessel leukocytoclastic vasculitis mainly characterized by purpura, arthralgia, abdominal pain, and nephritis, and is the most common vasculitis in children. Henoch–Schönlein purpura nephritis (HSPN) is one of the most important complications of IgA vasculitis, occurring in 20%–80% of patients with IgA vasculitis [1–4] and accounting for 1%–2% of all cases of childhood kidney failure [5, 6]. Nevertheless, only a few national epidemiological surveys have been conducted on the incidence rates of childhood HSPN worldwide and the results were varied. The lack of uniform diagnostic criteria for the diagnosis of “nephritis”—some studies consider hematuria to be “nephritis”, while others consider only kidney biopsy-proven “nephritis”–may explain why the incidence of HSPN varied among the different reports. Most studies only included HSPN diagnosed clinically in the presence of urinary findings, leading to an overestimation of the incidence of HSPN, and it remains controversial whether all of these findings are clinically significant. For these reasons, accurate epidemiological information is unavailable.

HSPN involves a wide variety of processes. The majority of pediatric HSPN patients have mild disease that improves spontaneously without treatment, and thus HSPN is considered a disease with a relatively good prognosis. However, 15% of patients with persistent hematuria and heavy proteinuria or with acute nephritis syndrome, 40% of patients with nephrotic syndrome, and 50% of patients with acute glomerulonephritis and nephrotic syndrome progress to chronic kidney disease [7]. Wakaki et al reported 45% of the patients with nephrotic state lasting for more than 3 months had unfavorable outcome, end-stage kidney disease or death [8]. However, the kidney biopsy indications and treatment protocols vary among institutions, there is no international consensus for evidence-based recommendations concerning the diagnosis and treatment of HSPN in children worldwide, and there are no diagnostic or treatment guidelines for HSPN in Japan.

The aims of the present study were to estimate the incidence of biopsy-proven HSPN and to investigate the biopsy criteria and treatment protocols in individual institutions in Japan to clarify the actual medical treatment situations for HSPN children. Knowledge on the epidemiology of HSPN is vital to help clinicians understand the extent of the disease and ultimately to improve its management.

## Materials and methods

### Study design and population

This cross-sectional nationwide institutional study was conducted by the Japanese Study Group of Renal Disease in Children. In December 2018, the survey was sent to 412 institutions throughout Japan, including all university and children’s hospitals, and all institutions that were members of the Japanese Society of Pediatric Nephrology. We selected these types of hospitals because children with apparent HSPN diagnosed by biopsy are usually referred to institutions meeting one of these criteria.

The survey consisted of a patient survey and an institutional survey.　 First, the patients survey was conducted on patients who were newly diagnosed with HSPN by kidney biopsy at the age of 1 year to 15 years between 01 January 2013 and 31 December 2015. The questionnaire was designed to record date of birth, date of biopsy, and sex as patient information of biopsy-proven HSPN during the study period. Next, we conducted an institutional survey in order to confirm the management policies of HSPN in each institution which responded that they perform kidney biopsy at their institutions. The criteria for kidney biopsy, methods of histopathological evaluation, treatment protocol, type of immunosuppressive drugs, treatment period, and protocol for re-biopsy were investigated.

### Definitions

The definitions used in the study were based on the American College of Rheumatology and The European League Against Rheumatism, the Pediatric Rheumatology International Trial Organization, and the Pediatric Rheumatology European Society criteria for IgA vasculitis published in 2010 [9]. Diagnosis of IgA vasculitis was based on purpura or petechiae as the mandatory criterion and the presence of one or more of the following criteria: acute-onset abdominal pain, arthritis or arthralgia, kidney involvement, hematuria (red blood cell count >5 red blood cells/high power field), and proteinuria (urinary protein-to-creatinine ratio (uTP/Cr) >0.3 g/gCr in spot morning sample). HSPN met the definition of IgA vasculitis, and was further defined by IgA-dominant immune deposits on immunofluorescence staining in kidney pathology findings.

Kidney dysfunction was defined as two-fold elevation from the basal Cr value. HSPN was divided into three groups based on the disease severity in the present study: severe HSPN was defined as ISKDC class IV or V and/or kidney dysfunction; mild HSPN was defined as ISKDC class I, II, or IIIa and uTP/Cr <1.0 g/gCr; and moderately severe HSPN was defined as those not included in severe or mild HSPN.

### Ethics

The study was conducted in accordance with the ethical principles of the Declaration of Helsinki and the ethical guidelines for Medical and Health Research Involving Human Subjects issued by the Ministry of Health, Labour and Welfare in Japan. The study was approved by a central ethics board at Tokyo Metropolitan Children’s Medical Center (approval number: H30b-129) before its commencement. Because all data were reported in a retrospective manner using patient charts, informed consent was not obtained in accordance with the above guidelines. Permission has been obtained from the Research Ethics Committee that consent does not need to be obtained from the parents or guardians of minors participating in the research since this is a retrospective research study. Study and participation details were posted on the institution’s official website with opt-out to disclose information about the implementation of the research, including the purpose of the research, so the patients can refuse to be the subjects of the research.

### Statistical analyses

Estimation of the number of patients who were newly diagnosed with HSPN by biopsy from the reported number of patients in our survey was conducted as follows. The reported number of patients in the survey was stratified by institution type (university hospital, children’s hospital, general hospital) and number of beds (<200, 200–500, ≥500).

Based on the assumption that the response rate was independent of the number of patients in each stratified category, the number of reported patients in each category was divided by the response rate and summed to calculate the total number of patients. The total number of patients was divided by the size of the population at risk in Japan reported by the Statistics Bureau of the Ministry of Internal Affairs and Communications of Japan (http://www.stat.go.jp/english/index.htm) to calculate the incidence of biopsy-proven HSPN in Japan.

## Results

### Basic characteristics and estimated incidence of biopsy-proven HSPN

A total of 353 of the 412 institutions (85.7%) responded to the questionnaire. Of the 353 institutions, 174 reported that they perform kidney biopsies at their institutions.

#### The indications for kidney biopsy for pediatric patients with HSPN in each institution

Since subjects of this study were patients with biopsy-proven HSPN, the indications for kidney biopsy at each institution are described first. Table 1A and 1B show the results of the responses regarding the time from clinical diagnosis of HSPN to kidney biopsy, which were obtained from each center, divided by the presence or absence of kidney dysfunction. The following characteristic points were noted. Patients with kidney dysfunction underwent biopsy relatively early, even if urinalysis showed uTP < 0.3 g/gCr. Meanwhile, in patients without kidney dysfunction, the timing for biopsy was determined by the amount of proteinuria; for example, 66.9% of patients with hypoalbuminemia (Alb <3.0g/dL) underwent biopsy within 1 month, while 68.2% of patients with 0.3 g/gCr ≤ uTP/Cr < 0.5 g/gCr of proteinuria were followed up for at least 6 months before biopsy.

**Table 1:** Indications for kidney biopsy and times to biopsy.

a. With kidney dysfunction							
	<1 mo	<2 mo	<3 mo	<6 mo	<1 yr	Not performed	n =
1. hypoalbuminemia (Alb <3.0 g/dL)	91.9%	1.3%	3.1%	1.3%	0%	0%	160
2. 1.0 g/gCr ≤ uTP/Cr	86.0%	2.5%	7.0%	1.9%	0%	0%	157
3. 0.5 g/gCr ≤ uTP/Cr < 1.0 g/gCr	68.2%	6.5%	14.8%	5.8%	1.3%	0%	155
4. 0.3 g/gCr ≤ uTP/Cr < 0.5 g/gCr	61.5%	7.2%	16.3%	8.5%	3.3%	0%	153
5. uTP/Cr <0.3 /gCr	43.7%	6.0%	17.2%	9.9%	4.0%	7.3%	151
b. Without kidney dysfunction							
	<1 mo	<2 mo	<3 mo	<6 mo	<1 yr	Not performed	n =
1. hypoalbuminemia (Alb <3.0 g/dL)	66.9%	11.5%	15.9%	2.5%	0.6%	0.6%	157
2. 1.0 g/gCr ≤ uTP/Cr	22.4%	13.0%	38.5%	20.5%	2.5%	0%	161
3. 0.5 g/gCr ≤ uTP/Cr < 1.0 g/gCr	3.2%	3.2%	32.1%	39.7%	14.7%	5.1%	156
4. 0.3 g/gCr ≤ uTP/Cr < 0.5 g/gCr	0%	0.6%	10.4%	32.5%	35.7%	16.2%	154
5. uTP/Cr <0.3 /gCr	0%	0%	0%	0.6%	1.2%	94.4%	162

Kidney dysfunction: Cr ≥2-fold the age reference value; mo: month; yr: year; Alb: albumin; uTP/Cr: urine protein-to-creatinine ratio.

#### Basic characteristics of biopsy-proven HSPN

A total of 353 institutions provided data for 563 children and adolescents aged from 1 year to 15 years who were initially diagnosed with HSPN by kidney biopsy.

The basic characteristics of children who underwent kidney biopsy for the above indication and were diagnosed with HSPN are shown in Table 2. Approximately 70% of the children were diagnosed at 4–9 years of age.

**Table 2:** Patient characteristics.

	Males	Females	All patients
Age distribution			
2 years	1 (0.3)	5 (1.9)	6 (1.1)
3 years	13 (4.3)	10 (3.9)	23 (4.1)
4 years	21 (6.9)	27 (10.4)	48 (8.5)
5 years	52 (17.1)	40 (15.4)	92 (16.3)
6 years	43 (14.1)	43 (16.6)	86 (15.3)
7 years	42 (13.8)	28 (10.8)	70 (12.4)
8 years	36 (11.8)	20 (7.7)	56 (9.9)
9 years	28 (9.2)	17 (6.6)	45 (8.0)
10 years	18 (5.9)	12 (4.6)	30 (5.3)
11 years	10 (3.3)	12 (4.6)	22 (3.9)
12 years	9 (3.0)	13 (5.0)	22 (3.9)
13 years	13 (4.3)	13 (5.0)	26 (4.6)
14 years	7 (2.3)	11 (4.2)	18 (3.2)
15 years	11 (3.6)	8 (3.1)	19 (3.4)
Total	304	259	563
Institution			
Children’s hospital	98 (32.2)	86 (33.2)	184 (32.7)
University hospital	123 (40.5)	107 (41.3)	230 (40.9)
General hospital	83 (27.3)	66 (25.5)	149 (26.5)

Data are shown as number (percentage) of patients

#### Estimated incidence of biopsy-proven HSPN

**Fig 1:**
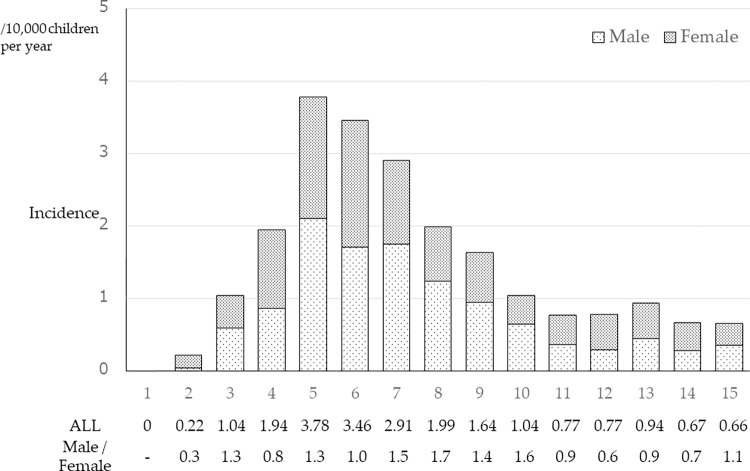
Estimated incidence of HSPN and male-to-female ratio. Estimated incidence of HSPN and male-to-female ratio in each age group of Japanese children aged 1 year to 15 years.

The response rates stratified by institution type and size are shown in Table 3. The questionnaire recovery rate was 85.7%, and 563 cases were reported per 3 years; per year, 188 cases of HSP diagnosed by kidney biopsy in this research. Considering a population of 16.4 million children aged 1 to 15 years in JAPAN, the collection rate and using the statistical methods described in the Statistical Analyses section, the estimated incidence of biopsy-proven HSPN in Japan was 1.32 (1.21–1.57) cases/100,000 children per year. The estimated annual incidence was highest between the ages of 5 and 7 years, and the median age at biopsy was 7.0 years (interquartile range: 5.0–9.0 years). The estimated incidence in males and females was 1.42 (1.24–1.60) and 1.22 (1.06–1.38)/100,000 children per year, respectively, with a male-to-female ratio of 1.2:1. The estimated incidence and male-to-female ratio in each age group are shown in Fig 1.

**Table 3:** Response rates according to size and type of institutions.

	Children’s hospitals	University hospitals	General hospitals
<200 beds	6/6 (100.0)	5/6 (83.3)	35/42 (83.3)
200–499 beds	12/14 (85.7)	17/24 (70.8)	112/130 (86.2)
≥500 beds	15/16 (93.8)	78/90 (86.7)	73/84 (86.9)
Total	33/36 (91.7)	100/120 (83.3)	220/256 (85.9)

Data are presented as number of responses/total number of institutions (percentage) in each category

### Management policies at individual institutions

#### Basis for histopathological evaluation

Our study revealed that 93.3% of institutions used the International Study of Kidney Disease in Children (ISKDC) criteria to assess HSPN pathology, followed by 7.1% for the Oxford classification that predicts outcomes based on histologically identical IgA nephropathy.

#### Treatment protocols for HSPN

A summary of the treatment protocols is provided in Table 4. Despite large variation in the treatment protocols for the three severity groups (for example, 40 different treatment plans for severe cases, 39 for moderately severe cases, and 17 for mild cases) at individual institutions, there were certain commonalities. Severe cases frequently received methylprednisolone pulse therapy with prednisolone and immunosuppressants, and moderately severe cases mostly received prednisolone and immunosuppressants. Mild cases most often received renin-angiotensin system inhibitors alone.

**Table 4:** Treatment protocols for HSPN in individual institutions.

	Severe	Moderately severe	Mild
With MPT	75.5%	26.2%	5.9%
With PSL	94.3%	90.5%	43.6%
With immunosuppressants	87.0%	77.6%	25.0%
Only RAS inhibitors	0.0%	6.2%	47.7%

MPT, methylprednisolone pulse therapy; PSL, prednisolone; RAS, renin-angiotensin system

Severe HSPN was defined as ISKDC class IV or V and/or kidney dysfunction, mild HSPN was defined as ISKDC class I, II, or IIIa and UTP/Cr <1.0 g/gCr, and moderately severe HSPN was defined as those not included in severe or mild HSPN.

The definitions of HSPN severity were as follows: severe HSPN was defined as ISKDC class IV or V and/or kidney dysfunction; mild HSPN was defined as ISKDC class I, II, or IIIa and UTP/Cr <1.0 g/gCr; and moderately severe HSPN was defined as those not included in severe or mild HSPN.

Treatments were selected from following options: a) PSL alone; b) PSL + immunosuppressants; c) multiple drug therapy (including PSL, immunosuppressants, warfarin, dipyridamole); d) MPT; e) RAS inhibitors; f) tonsillectomy; g) urokinase pulse therapy; h) plasma exchange; i) no treatment

More than 75% of severe and moderately severe HSPN cases were treated with immunosuppressants; mizoribine was the most commonly selected in 71.3% of institutions followed by cyclosporine in 18.6% and azathioprine in 11.4%. Meanwhile, 4.8% of institutions selected oral cyclophosphamide, intravenous cyclophosphamide, and mycophenolate mofetil, respectively.

Fifty-seven percent of institutions continued treatment for 2 years, and 23.8% of other institutions until remission was achieved. Meanwhile, 19.4% of them selected other endpoints, such as continuation of prednisolone for 6 months or 1 year regardless of the urine findings. Based on the present results, most institutions chose to continue some type of medication for 2 years.

#### Re-biopsy after treatment

The decision on whether to perform a kidney re-biopsy after treatment depended on whether the patients were in remission. If patients achieved remission and had no abnormalities on urinalysis after treatment, only 17.8% of institutions performed a re-biopsy. Meanwhile, if abnormal urinary findings remained, 77.4% of institutions performed a re-biopsy.

## Discussion

We conducted the first nationwide epidemiological survey of biopsy-proven HSPN in Japan, with a population of 16.4 million children aged 1 to 15 years. A relatively high response rate of 85.5% was obtained, suggesting that the results of the study adequately reflect the current epidemiology of biopsy-proven HSPN in Japan. The study revealed that the estimated incidence of pediatric biopsy-proven HSPN in Japan was 1.32 cases/100,000 children per year. The male-to-female ratio was 1.2:1, and approximately 70% of the children were diagnosed at 4 to 9 years of age.

Reports on the estimated incidence of HSPN are very scarce. As far as we could determine, two nationwide surveys and one local survey have been reported. The annual incidence of HSPN was 2.7 cases/100,000 children per year in England [10], 1.9 cases/100,000 children per year in Italy [11], and 3.5–3.6 cases/100,000 children per year in Fukushima, Japan [12]. The estimated incidence of HSPN in the present study was 1.32 cases/100,000 children per year, which is lower than the findings in the previous reports, and may have arisen because the study only included biopsy-proven HSPN cases. A kidney biopsy is basically performed in all cases requiring treatment. Thus, the study is considered very significant because it was possible to clarify the frequency of HSPN requiring treatment by targeting cases undergoing kidney biopsy. Furthermore, the study included only cases with biopsy-confirmed HSPN. We think that these are strengths of the present study. The predominant age was reported to be 4 to 7 years in the past reports [18–20], which was almost the same as the results of the present study. The male-to-female ratio for childhood HSPN varied among different studies. Consistently, however, most of the series described a higher incidence in males (ratio ranging from 1.5 to 1.7) [13, 14]. In the present study, the male-to-female ratio was not notable, and the difference was considered to arise from racial differences and genetic backgrounds.

We also investigated the indications for kidney biopsy at individual institutions to confirm that the calculated morbidity was accurate. There is no international consensus for evidence-based recommendations concerning the indications for kidney biopsy and treatment of HSPN in children. In our results, patients with kidney dysfunction underwent biopsy relatively early even if their urinalysis showed mild proteinuria (uTP/Cr <0.3 g/gCr). Meanwhile, the indications for biopsy in patients without kidney dysfunction were determined by the amount of proteinuria. Despite certain commonalities, each institution had their own specific indications for kidney biopsy. The SHARE recommendations for the diagnosis of IgA vasculitis should be noted [3]: a kidney biopsy should be performed if an IgA vasculitis patient has severe proteinuria (>250 mg/mmol for at least 4 weeks; although shorter duration of severe proteinuria is also a relative indication for biopsy), persistent moderate (100–250 mg/mmol) proteinuria, or impaired glomerular filtration rate. The recommendations specify the criteria for kidney biopsy, but do not state the period until a kidney biopsy is performed unless severe proteinuria persists.

There is no evidence-based consensus for which histological evaluation should be used to assess the severity of HSPN and its prognosis. Ninety-three percent of institutions used the ISKDC classification to estimate the pathology of HSPN in the present study. The kidney biopsy findings in HSPN are essentially indistinguishable from those in idiopathic IgA nephropathy. A consensus on the pathologic classification of IgA nephropathy was developed by the International IgA Nephropathy Network in collaboration with the Renal Pathology Society [15, 16], and the Oxford classification of IgA nephropathy is now the consensus pathologic classification used worldwide. The ISKDC classification is currently the most commonly used pathological grading system in HSPN patients, but there is a lack of consensus regarding the utility of the ISKDC classification as a predictor of long-term prognosis. [17, 18]. In recent years, evaluation of HSPN using the Oxford classification was reported to be useful [19, 20]. Yun et al. [20] reported a correlation between kidney histopathology at disease onset and long-term prognosis when patients with pediatric HSPN were evaluated by the Oxford classification. We are currently planning to conduct a secondary survey including detailed case examinations and assessments of kidney histopathological specimens from the same patients by both the ISKDC classification and the Oxford classification, as well as evaluation of the clinical courses, especially the prognosis. In the future, new predictors for the kidney prognosis of pediatric HSPN patients may be established and the usefulness of pathological findings may be improved.

No one therapy has proven to be of value in the treatment of HSPN and, while our results demonstrated certain commonalities in treatment protocols, there was marked variation in the specific protocols used among the institutions who took part in this study. Although several regimens, including glucocorticoids, immunosuppressants, and plasma exchange, have been reported and tested with controversial results [21], there is no evidence for the use of oral glucocorticoids in HSPN. Data from randomized controlled studies on patients with IgA nephritis demonstrated a benefit in reducing proteinuria, and the KDIGO Clinical Practice Guidelines for Glomerulonephritis proposed the same treatment for IgA nephritis and HSPN when the clinical conditions were similar. However, the guidelines assigned low levels of evidence for almost all recommendations and suggestions related to HSPN. Nevertheless, HSPN and IgA nephropathy are thought to share a common pathology, however they have different clinical features and prognoses, so no conclusion has been reached as to whether they are the same or different disease. However, they clearly have different clinical features and prognosis in many cases. Therefore, if useful treatments specific for HSPN can be clarified, the prognosis of HSPN can be improved.

Our study has several limitations. First, because the study limited the facilities receiving the questionnaire to all university and children’s hospitals, and all institutions that were members of the Japanese Society of Pediatric Nephrology, the number of patients may be underestimated. However, it is considered that kidney biopsy and treatment for severe HSPN are rarely performed at institutions without pediatric nephrologists, and thus the present study has clarified the incidence of HSPN relatively accurately in Japan. Second, the incidence of HSPN may be underestimated because we investigated only biopsy-proven HSPN cases and the indications for kidney biopsy varied among the facilities. On the other hand, we believe that this study is very significant in that we are able to determine the frequency of HSPN requiring treatment by targeting cases undergoing kidney biopsy and only including cases with biopsy-confirmed HSPN. The severity of HSPN varies widely, ranging from spontaneous improvement without treatment to kidney failure. In severe cases that require treatment, a kidney biopsy is performed to evaluate the kidney pathology severity, and we believe this is the first report to clarify the frequency of pediatric HSPN cases that require such intervention. In addition, the diagnosis of HSPN is usually made by clinical symptoms in the outpatient setting and is not based on uniform criteria in individual institutions. The addition of IgA deposition by immunofluorescence staining in kidney pathology to the diagnostic criteria will make it possible to recruit patients diagnosed according to uniform criteria.

## Conclusions

In conclusion, based on the results of our nationwide survey, we estimated the incidence of biopsy-proven HSPN in Japan to be 1.32 cases/100,000 children per year. Although the age at onset was similar to that for children in previous studies. From institutional study, biopsy indications and treatment protocols varied by institution. Although most HSPN cases are considered to be self-limiting and to spontaneously resolve without treatment, some cases progress to end-stage kidney disease even with treatment. An evidence-based treatment protocol needs to be established to improve the prognosis of potentially exacerbated cases. Further studies are warranted to establish an optimal treatment policy based on the prognosis. We are currently conducting a detailed case study based on the same cohort to investigate detailed clinical courses, pathological findings, and the long-term courses of HSPN in Japanese patients.
